# Evaluation of Bioaerosol Bacterial Components of a Wastewater Treatment Plant Through an Integrate Approach and In Vivo Assessment

**DOI:** 10.3390/ijerph17010273

**Published:** 2019-12-30

**Authors:** Erika Bruni, Giulia Simonetti, Beatrice Bovone, Chiara Casagrande, Federica Castellani, Carmela Riccardi, Donatella Pomata, Patrizia Di Filippo, Ermanno Federici, Francesca Buiarelli, Daniela Uccelletti

**Affiliations:** 1 Department of Biology and Biotechnology “C. Darwin”, Sapienza University of Rome, 00185 Rome, Italy; erikabruni89@gmail.com (E.B.); beatricebovone@gmail.com (B.B.); 2 Department of Chemistry, Sapienza University of Rome, 00185 Rome, Italy; giulia.simonetti@uniroma1.it (G.S.); federica.castellani@uniroma1.it (F.C.); ca.riccardi@inail.it (C.R.); d.pomata@inail.it (D.P.); p.difilippo@inail.it (P.D.F.); francesca.buiarelli@uniroma1.it (F.B.); 3 Department of Chemistry, Biology and Biotechnology, University of Perugia, 06123 Perugia, Italy; chiara.casagrande@hotmail.it (C.C.); ermanno.federici@unipg.it (E.F.); 4 Inail DIT, 00143 Rome, Italy

**Keywords:** airborne, *Caenorhabditis elegans*, worker exposure, bacterial bioaerosol, wastewater

## Abstract

Wastewater carries different pathogenic and non-pathogenic microorganisms that can be dispersed in the surrounding environment. Workers who frequent sewage treatment plants can therefore be exposed to aerosols that contain a high concentration of potentially dangerous biological agents, or they can come into direct contact with contaminated material. This can lead to allergies, infections and occupational health-associated diseases. A characterization of biological risk assessment of bioaerosol exposure is necessary. The aim of this study was to evaluate the application of an interdisciplinary method that combines chemical and biological approaches for the analysis of a bioaerosol derived from a wastewater treatment plant (WWTP) situated in Italy. Sampled filters were analyzed by HPLC-MS/MS spectroscopy that searched for different chemical biomarkers of airborne microorganisms. The analytical quantification was compared to the biological cultural method that revealed an underrated microbial concentration. Furthermore, next generation sequencing analysis was used also to identify the uncultivable species that were not detected by the culture dependent-method. Moreover, the simple animal model *Caenorhabditis elegans* was used to evaluate the pathogenicity of two isolates—*Acinetobacter iwoffii* and *Micrococcus luteus—*that showed multidrug-resistance. This work represents a starting point for the development of a multidisciplinary approach for the validation of bioaerosol exposure on WWTP workplaces.

## 1. Introduction

Bioaerosols are composed of particles of biological origin that are suspended in a gaseous medium in which there is the presence of not only fungi and bacteria but also viruses, pollens, plant debris, and their byproducts such as endotoxins, allergens, and mycotoxins [1]. Nowadays, great interest is given to bioaerosol evaluation in different fields, starting from the preservation of cultural heritage to occupational environments [2,3]. In fact, it is necessary to assess the danger of workplace breathable air in order to protect the health of those who work there as much as possible, preventing the onset of chronic and dangerous diseases. Wastewater treatment plants (WWTP) can represent a hazard for the environment, for the operators and the surrounding population due to the risk that is associated with exposure to biological agents [4] that are aerosolized during the different phases of the process.

It is well known that the aerosolization of drops that are produced by the bursting of bubbles causes the generation of bioaerosol [5], which may contain many pathogenic microorganisms whose concentration and propagation are influenced by different parameters such as the meteorological conditions (wind speed, humidity, temperature, etc.) [6,7].

Assessments of the hazard of bioaerosol exposure have usually focused on identifying the aerosolized pathogens that are produced in the aeration basins from WWT, such as *Klebsiella pneumoniae*, *Legionella* sp., *Acinetobacter* sp. and *Mycobacterium tuberculosis*, producing a serious hazard to on-site operators and adjacent communities [8,9,10,11,12,13]. Furthermore, WWTPs are the main source of antibiotic releases in the environment, and they contribute to the dissemination of antibiotic resistance genes (ARGs) among bacteria, many of which are pathogenic to humans [14,15,16]. Bacteria can acquire more resistance genes through horizontal gene transfer from other bacteria and through spontaneous mutation, becoming multidrug-resistant or “superbugs.” Nowadays, antibiotic-resistant bacterial infections that are acquired during specific work activities can be considered an integral part of occupational biological risk [17].

Normal operations that take place at WWTPs (the aeration and mechanical agitation of raw wastewater) generate different pathogenic and non-pathogenic microorganisms and can endanger human health through inhalation, contact, and ingestion [5]. Fungal spores and bacteria have a typical aerodynamic diameter (Da) of about 1.5–30 and 1–3 μm, respectively [18], and due to their small size, they become a potential cause of infections in immune-compromised people once inhaled and may cause allergic responses [19]. Most studies on bioaerosol have traditionally been conducted by collecting and analyzing fungal spores and bacterial cells with total-count and culture techniques [20,21,22,23]. However, since the end of nineties, it has been estimated [24,25,26] that only 1.5%–15.3% of airborne microbial cells can be enumerated by the direct counting of the colonies (viable microorganisms) after incubation on Tryptic Soy Agar (TSA) [27]. The remaining species, which are viable but non-cultivable (VNC)—because of intra-cellular stress or to culture medium or incubation temperature—or non-viable (NV), cannot be identified. More recent studies in literature have focused on the DNA-based detection systems, allowing for all the microorganisms (live, dead, and VNC) to be examined regardless of the ability of those cultivating them [9,28,29,30,31]. In addition, the culture methods for detecting these pathogens in certain “bio-rich” samples derived from WWTP environments can be misleading, since they could be hampered or hidden by the fast and total growth of other microorganisms, such as molds. Plate counts promote fast-growing bacteria instead of slow-growing bacteria: A poor yield of colonies on solid media could be caused by factors, including media selection, incubation time, and growth conditions, beyond the high percentage of unculturable microorganisms [32,33]. Chemical markers can be considered as an integrative approach to microbiological and molecular biology techniques for the determination of atmospheric bioaerosol composition [34,35,36]. The basis of this approach is that bioaerosol components contain chemical compounds that can be used as markers of larger and/or bioactive structures. Therefore, they can be regarded as molecules that, with their relevance, confirm the presence of a certain target that can be measured. In this case, the chosen molecular targets reveal the biological nature of the airborne particles.

In this work, a multidisciplinary approach—biological and chemical—was applied to bioaerosols collected in a WWTP. The chemical markers chosen were muramic acids for bacterial cells, dipicolinic acid for bacterial spores, and ergosterol for fungal spores [37,38]. We sampled near the aeration tanks for biological treatment where the concentration of bioaerosols was generally expected to be high. An investigation of bioaerosols at work places should focus not only on the characterization of airborne microorganisms but also on their multidrug-resistance. In this work, two of bacterial pathogens isolated from the WWTP bioaerosols were examined for antibiotic susceptibility by agar diffusion. Moreover, the possible pathogenicity of the two isolated bacteria was tested by using the *Caenorhabditis elegans* model system, a powerful in vivo tool for studying host–pathogen interactions. Its vitality, larval development, progeny, and neuro-muscular behaviors represent pathogenicity markers [39].

## 2. Materials and Methods

### 2.1. Sampling

This study was performed at a wastewater treatment plant (named WWTP_RE) located in central Italy, East Rome (RE). This plant was mainly composed of the following stages: coarse screening; primary settling; biological processing, which allows for the removal of the organic pollutants load; secondary settling, and a disinfection process to obtain an effluent that is suitable to be released into the environment. The sampling campaign was carried out at WWTP_RE in Spring 2018 with an average temperature of 15 °C. Usually, the mild temperature, characterizing Rome during the spring season, promotes bacterial growth, according to Brągoszewska et al. [40]. This campaign consisted of two sequential samplings. Table 1 shows the details of the two sampling campaigns.

Two DLPI (Dekati^®^ Low Pressure Impactor, Dekati Italia, Albiate MB, Italy) 13-stage cascade impactors, operating at a flow rate of 10 L/min, were used in parallel to sample particles in the range of 0.03–10 μm on 25 mm polycarbonate collection substrates (Merck Millipore, Merck spa, Vimodrone, MI, Italy) [36,41,42,43]. Before each sampling, the filters and all used tools were sterilized by a UV lamp.

**Figure 1:**
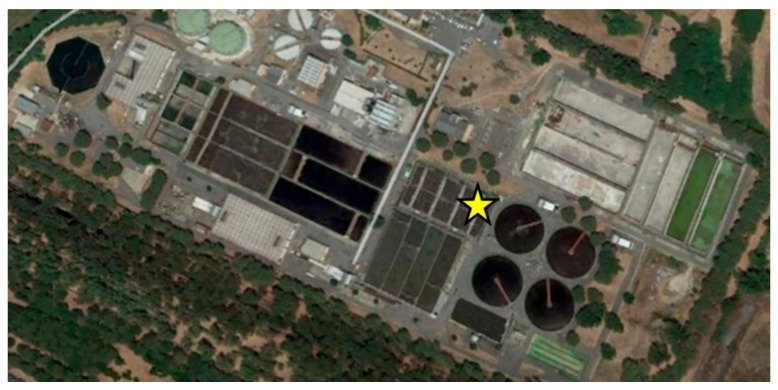
Wastewater treatment plants with the oxidation aeration tanks highlighted by the yellow star.

The samplers were placed next to the aeration tanks under a 3 m high shelter at about 1.50 m from the floor (Figure 1).

On the first and last day of the sampling campaign, plates that contained specific culture media were used for the collection of bacteria and fungi.

Finally, during the two monitoring campaigns, field blanks were collected for the whole duration of each single sampling.

### 2.2. Experimental

Chemicals, materials and instrumentation are reported in the following Table 2 and Table 3.

### 2.3. Sample Preparation for Chemical Analyses

For chemical analyses, the thirteen filters of the first multistage impactor were re-assembled into two sets that corresponded to particle fraction with Da < 1 µm (particulate matter (PM) < 1) and Da > 1 µm (PM > 1). Sample preparation was extensively detailed in a previous paper [44]. Shortly after sampling, filters were added to 500 µL of deionized water. To break the cell walls, the samples underwent seven freezing (−20 °C) and thawing cycles, with glass beads, stirring in ultrasound, and vortexing after each cycle. After the addition of iso-octane (ISO) (1:1), the two layers (aqueous and organic) were separated and singularly analyzed for the detection of dipicolinic and ergosterol, respectively, by HPLC-ESI-MS-MS and HPLC-APCI-MS-MS analyses.

The successive hydrolysis of the aqueous fraction with 6% HCL at 100 °C for 3 h, followed by neutralization with NH_3_ and purification through a solid phase extraction C_18_ cartridge, allowed for the detection of muramic acid by HPLC-ESI-MS-MS analysis. The limit of detection (LOD) of the method was determined by spiking blank filters with the analyte before the whole procedure. The concentration of the injected analyte that produced a peak with a signal-to-noise ratio (S/N) of 3 was chosen as the LOD. The limit of quantification (LOQ) was estimated in the same way as the LOD—with the use of a criterion (S/N) of 10. All the validation parameters were evaluated in our previous investigation [44].

### 2.4. Isolation of Cultivable Bacteria

The determination of the vital species was carried out on each of the thirteen filters sampled with the second multistage impactor through standard microbiological methods for the analysis of cultivable microorganisms.

The isolation of the cultivable microorganisms was achieved by washing each filter in a sterile 0.05% Tween80 solution (Sigma-Aldrich, St. Louis, MO, USA) under agitation (150 rpm). The washing solutions were then serially diluted, and aliquots of these dilutions were plated both on plates of Difco Nutrient Agar and Difco yeast extract peptone dextrose (YPD; yeast extract 10 g/L, peptone 20 g/L, dextrose 20 g/L). Empty filters were considered as controls. A first count of the obtained single colonies (CFU—colony forming unit) was made after 24 h of incubation at 28 °C and subsequently updated at 72 h.

### 2.5. DNA Extraction and Bacterial Isolates Identification

Bacterial DNA was obtained from isolated colonies by microLYSIS (Microzone Ltd., Haywords Heath, W. Sussex, UK) according to the manufacturers’ instructions, and this DNA was used as template for PCR amplifications. Universal primers F16S-8 (5′-AGAGTTTGATCCTGGCTCAG-3′) and R16S-1492 (5′-GGTTACCTTGTTACGACTT-3′) (Sigma-Aldrich) were used to amplify the eubacterial 16S rDNA segments. The PCR amplification reaction was performed by using the Biometra T-Personal Thermocycler (Analytic Jena, Jena, Germany) at a final volume of 25 μL. Every mixture contained 1 μL of microlysis, 1 mM dNTP Mix (Bioline, London, UK), 0.2 µM/each forward and reverse primer, 2 mM MgCl_2_, and 1.25 U Accuzyme DNA polymerase (Bioline) in the supplied buffer. The thermo-cycling conditions were as follows: 95 °C for 2 min, followed by 30 cycles of 95 °C for 30 s, 57 °C for 30 s, 72 °C for 2 min, and a final extension step of 72 °C for 5 min. Finally, 5 μL of each PCR sample were loaded on 1.5% agarose gel and controlled by electrophoresis.

PCR amplification products were ligated into the pGEM-T Easy cloning vector (Promega, Fitchburg, MA, USA), and ligation products were then transformed into *Escherichia coli* DH5α competent cells. Selected colonies arising from the transformation were picked out by blue/white screening and directly checked by colony PCR. Clones containing the correct-sized insert were grown up in Luria- Bertani (LB) broth with antibiotic selection, and plasmid DNA was extracted by using the alkaline lysis method [45]. The sequencing of plasmid inserts was realized by using the T7 primer (BMR Genomics Sequencing Service, Padova, Italy). The resultant DNA sequences of amplified 16S rDNA fragments were subjected to BLASTn analysis in order to get a taxonomic identification [46].

### 2.6. Metagenomic DNA Extraction and Analysis

The assessment of bacterial communities that are associated with particulate matter was carried out with a culture-independent approach based on the next generation sequencing of 16S rRNA gene fragments, as reported previously [47]. Metagenomic DNA was directly extracted from high-volume filters by cutting a 4 cm^2^ portion, in aseptic conditions, onto small pieces (approximately 2–3 mm^2^) and by using the FastDNA^®^ Spin Kit for Soil (MP Biomedicals, Solon, OH, USA). The manufacturer’s protocol was followed with the only exception that the homogenization step was repeated twice.

The V3–V4 hypervariable regions of the 16S rRNA gene were PCR-amplified by using the universal primers indicated by reference [48], and they were modified by adding external barcodes to permit the parallel processing of multiple samples. Multiplexed libraries were then sequenced with a 250 bp × 2 paired-end protocol in the MiSeq Illumina platform by Bio-Fab research srl (Roma, Italy). Sequencing reads were processed with the QIIME bioinformatic pipeline by merging forward and reverse reads, and they were quality filtered with Q > 30. Operational taxonomic units (OTUs) were defined by clustering the sequences at 97% sequence identity, eliminating those present with low abundances (<0.005%), and then these OTUs were assigned to taxonomic ranks by using the GreenGene database.

### 2.7. Determination of Antibiotic Susceptibility

Antibiotic susceptibility tests on bacterial isolates were realized with Biolab Zrt. (Budapest, Hungary) antibiotic discs through the Kirby–Bauer method as described by Schifano et al. [49]. Each disc of a 6 mm diameter contained the following antibiotics at a determined amount: amikacin (30 µg), aztreonam (30 µg), vancomycin (30 µg), streptomycin (25 µg), erythromycin (15 µg), tetracycline (30 µg), cefalotin (30 µg), gentamicin (10 µg), cefotaxime (30 µg), chloramphenicol (30 µg), clindamycin (30 µg), penicillin G (10 µg), ampicillin (10 µg), tobramycin (10 µg), cefuroxime (30 µg), oxacillin (1 µg), fosfomycin (50 µg), rifampicin (30 µg), carbenicillin (100 µg), and mezlocillin (75 µg). Briefly, 100 µL of bacterial over-night culture was spread on Nutrient-Broth (NB) agar plates where the antibiotic discs were later placed. Plates were incubated at 30 °C for 24–72 h. The inhibition zones were measured from the center of each disc and expressed in centimeters.

### 2.8. Caenorhabditis Elegans Growth and Maintenance

All the animals used in this work belonged to the N2 wild-type strain. In normal conditions, they grew at 16 °C on Nematode Growth Medium (NGM) agar plates seeded with an OP50 *Escherichia coli* suspension as a feeding source [50].

### 2.9. Nematodes Infection

Infection plates of isolated *Micrococcus luteus* NCTC2665 and *Acinetobacter iwoffii* ZS207 were prepared by spotting 30 μL of overnight culture grown in a nutrient broth on 35 mm NB agar plates. These plates were incubated overnight at room temperature (RT) and used for every infection assay.

Synchronized one-day-adult nematodes on OP50 *E. coli* were shifted to 25 °C for the infection. The worms that were collected from these plates were washed with a sterile 1× M9 buffer (KH_2_PO_4_ 3 g/L, Na_2_HPO_4_ 6 g/L, NaCl 5 g/L, and MgSO_4_ 1 mL 1 M) and subsequently placed on appropriate infection plates. This procedure was also done for the preparation of plates for lifespan, brood-size and body-length assays.

### 2.10. Life Span Analysis

About fifty one-day-adult animals derived from a synchronized cultures grown on NGM-OP50 plates at 16 °C were transferred onto NB agar infection plates and maintained at 25 °C for the entirety of the experiments. Fifty nematodes were always fed continuously with *E. coli* OP50 on NGM agar plates and used as controls. Every day, animals were monitored, counted, and moved to fresh plates, thanks to the help of an Optech SZ-N series stereomicroscope (Exacta+Optech, Modena, Italy). The nematodes were considered dead if they did not respond to mechanical stimulus from a small platinum wire. All the experiments were made in duplicate and repeated at least three times.

### 2.11. Nematodes Brood Size Assays

To assess the infection effects on reproduction, four fertile worms from the NGM-OP50 synchronous culture were washed and then separately transferred to NB infection plates daily. The eggs were counted until the end of the reproductive period.

### 2.12. Measurement of Nematodes Body Length

For the determination of the infection incidence on development and growth, nematode larvae that were infected with each bacterium, starting from embryos hatching, were photographed at the indicated time points by using a Leica MZ10F stereomicroscope with a Jenoptik CCD camera. The length of worm body was measured by using the Delta Sistemi IAS software. An average of 30 nematodes was imaged on at least three independent experiments.

### 2.13. Body Bend Analysis

Locomotion alterations were analyzed by measuring the body bend frequency of the nematodes fed with isolated bacteria, comparing them to the control ones [51]. In particular, nematodes were washed and placed in 10 μL of the M9 buffer, allowing them to swim freely: body bending was evaluated on 10 animals for conditions during a period of 60 s.

### 2.14. Pharyngeal Pumping Assay

The pharyngeal pumping rate of nematodes fed with different isolated bacteria was made as described in [52]. Three independent experiments were carried out with 30 worms gauged in each experiment.

### 2.15. Statistical Analysis

All experiments were performed in triplicate. The statistical significance was determined by Student’s *t* test or a one-way ANOVA analysis coupled with a Bonferroni post-test (GraphPad Prism 5.0 software, GraphPad Software Inc., La Jolla, CA, USA) and defined as * *p* < 0.05, ** *p* < 0.01, and *** *p* < 0.001. *Ns* is non-significant.

## 3. Results and Discussion

### 3.1. Bacterial Cells and Fungal and Bacterial Spores by Chemical Approach

**Figure 2:**
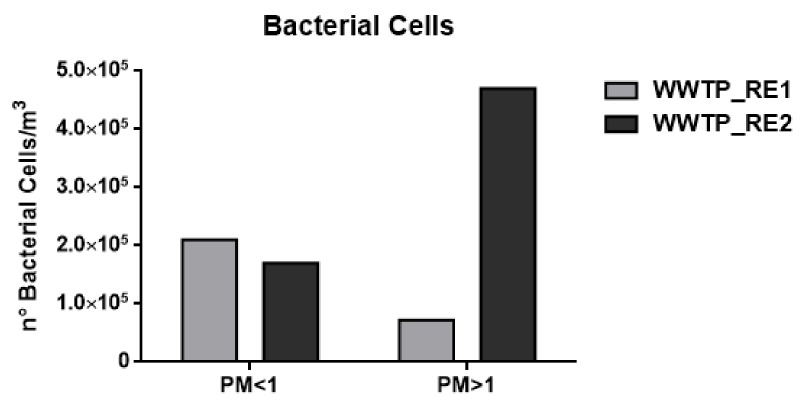
Number (n°) of total bacterial cell/m^3^ in the wastewater treatment plant (WWTP). Sampling campaign WWTP_RE1 (first period) and WWTP_RE2 (second period).

**Figure 3:**
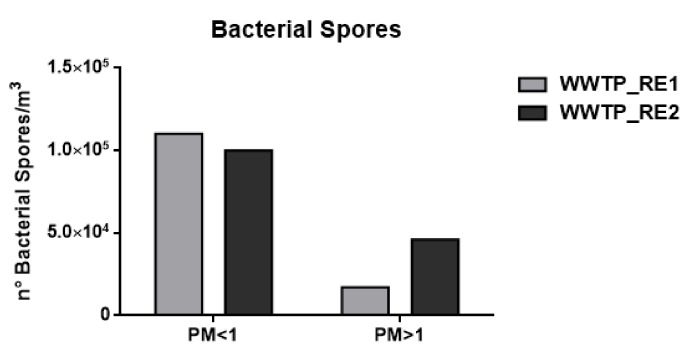
Number (°) of total bacterial spores/m^3^ in the wastewater treatment plant (WWTP). Sampling campaign WWTP_RE1 (first period) and WWTP_RE2 (second period).

Quantitative analyses of microbes were carried out by using matrix calibration curves to bypass eventual signal suppression [53]. Muramic acid was used as a biomarker of bacterial vegetative cells by applying a conversion factor of 0.6%, and dipicolinic acid was used as a biomarker of bacterial spores by applying a conversion factor of 10% [37,44]. Figure 2 and Figure 3 show the number of cells and spores/m^3^, assuming the average weight of a bacterial cell to be 1 pg and of a bacterial spore to be 0.6 pg.

Ergosterol was used as biomarker of fungal spores and the conversion factor of 3.2 ng of ergosterol (ERG)/µg spore was employed [54].

**Figure 4:**
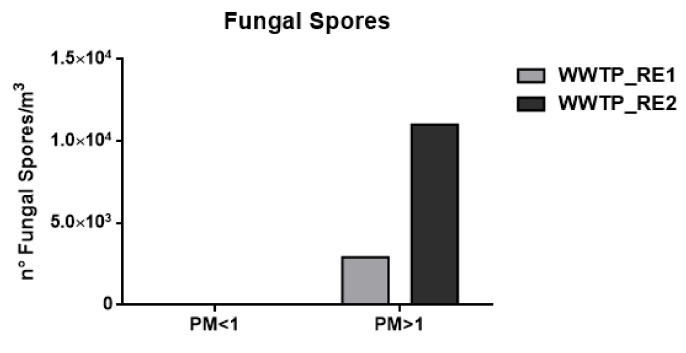
Number (n°) of total fungal spores/m^3^ in the wastewater treatment plant (WWTP). Sampling campaign WWTP_RE1 (first period) and WWTP_RE2 (second period).

Figure 4 shows the fungal contribution (number of spores/m^3^) calculated assuming the weight of fungal spore 65 pg [55].

**Figure 5:**
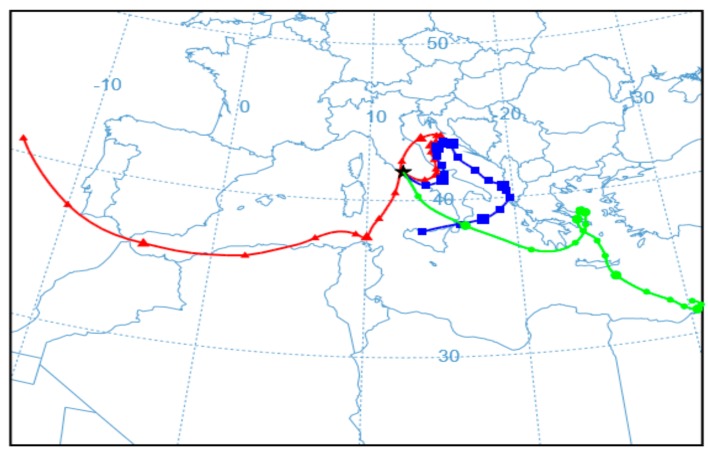
Simulation, according to the HYSPLIT model, of the wind trajectory coming from the desert areas of North Africa (16 April 2018 H 12:00).

As shown in Figure 2 and Figure 3 and Table 4, the concentration of bacterial cells and spores in WWTP_RE1 was found to be higher in PM < 1 fraction (fine fraction) compared to PM > 1 (coarse fraction); this could be explained by the fact that Saharian dust events induce a higher aggregation of PM. The dominating mode of small airborne bacteria in these sampling sites, especially close to oxidation tank, could be due to the emission of bacteria into the air in the form of single cells or small aggregates [56]. In WWTP_RE2, the bacterial spores showed a similar trend compared to the first sampling, whereas bacterial cells registered an opposite trend with highest level of concentration in the coarse fraction (PM > 1). In fact, the PM > 1 concentration of bacterial cell in WWTP_RE2 was six times higher than WWTP_RE1. The existing differences were due to an advection episode of air masses coming from the desert areas of North Africa—these masses hit Central Italy during those monitoring days (11–17 April). According to the literature, this type of event has a positive effect on bacterial growth and abundance. Microorganisms, mobilized into the atmosphere along with desert soil, are capable of surviving in a long-range transport on a global scale, inducing an increase of the bioaerosol level in the dust storm affected areas [57,58]. Figure 5 shows the back trajectory of the wind calculated by the HYSPLIT model between the 11th and 16th of April [59].

This event implies that the bacterial load was not completely related to the WWTP bioaerosol production in this campaign. However, the results proved to be comparable to those reported in previous studies, showing concentrations of around 10^5^ bacteria/m^3^ [60]. In the case of fungal spore concentrations, detectable values were only found in the coarse fraction, which in agreement with their characteristic aerodynamic diameter of 1.5–30 μm [18]. Besides bacteria, the increase of fungal spore concentration in WWTP_RE2 was also justified by the Saharan dust event.

As a control, an urban area near the plant was selected; the data showed a bioaerosol concentration that was eight times lower than the WWTP aerosol. For our purposes, this contribution was considered negligible (data not shown).

### 3.2. Bacterial Bioaerosol Diversity and Taxonomic Composition

**Figure 6:**
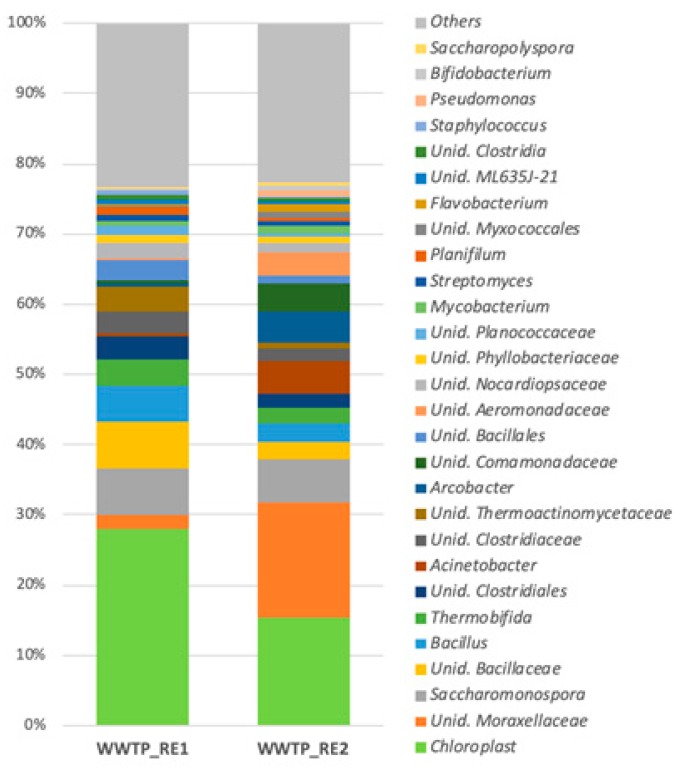
Most abundant genera (>1%) in WWTP_RE1 and WWTP_RE2 samples (Unid, unidentified).

A taxonomic analysis of the bacterial community was performed on both the WWTP_RE1 and WWTP_RE2 by the Illumina sequencing of 16S rRNA gene fragments. The results at the genus level of bacterial communities are illustrated in Figure 6, which reports the most abundant genera (>1% in at least one sample).

*Chloroplast* (27.9% and 15.4%, respectively), *Moraxellaceae* (2% and 16%, respectively), *Bacillus* (5.1% and 2.5%, respectively), and *Bacillaceae* (6.7% and 2.5%, respectively) were the main existing genera in both the WWTP_RE1 and WWTP_RE2 samples. On the other hand, *Acinetobacter* (0.6% and 4.5%, respectively), *Arcobacter* (0.3% and 4.3%, respectively), *Aeromonadaceae* (0.2% and 3.4%, respectively), and *Comamonadaceae* (0.5% and 4.1%, respectively) were found predominantly in the second sampling, maybe due to the Saharan dust event. The genera of *Saccharomonospora* and *Thermobifida* were present in both samples, although in low amounts (6% and 3%, respectively).

### 3.3. Isolation of Viable Bacteria

In the first and last day of each sampling, plates were used in triplicate for the collection of both bacteria and vital molds, respectively. The Petri dishes were left open for one hour near the sampling site in order to obtain an analysis of the viable microorganisms. The preliminary analysis showed a homogeneity between the quantity and the quality of the cultivable species present in the air near the sampling site, highlighting a greater concentration of bacterial charge compared to the fungal one (data not shown).

**Figure 7:**
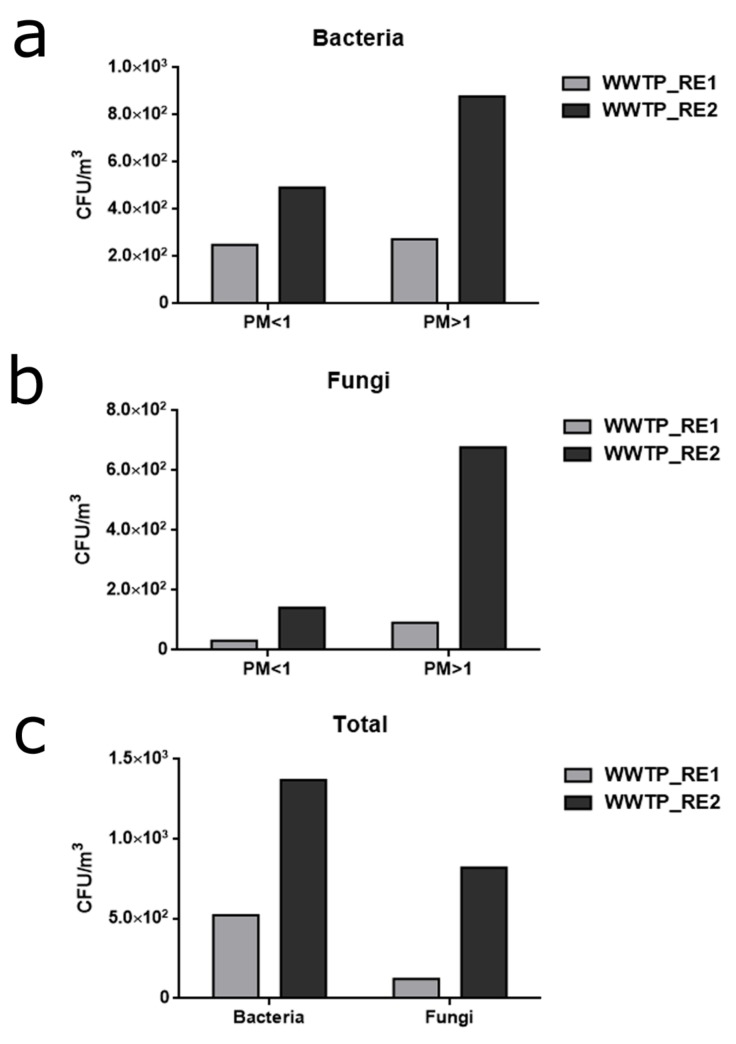
Cultivable microbial charge derived from the filters of the two subsequent samplings of WWTP_RE1 and WWTP_RE2. (**a**) Bacteria colony forming unit (CFU)/m^3^ obtained on NB agar plates by washing the sampled filters. Amounts for fine (PM < 1) and coarse fraction (PM > 1) are reported for each sampling. (**b**) Fungi CFU/m^3^ obtained on yeast extract peptone dextrose (YPD) agar plates by washing the sampled filters. Amounts for fine (PM < 1) and coarse fraction (PM > 1) are reported for each sampling. (**c**) Total amount of collected CFU/m^3^ of bacteria and fungi in the WWTP_RE1 and WWTP_RE2 samplings.

These data were in agreement with the CFU amount that was derived from the washing of the sampled filters (Figure 7c). Looking at Panels A and B of Figure 7, it can be seen that the number of CFUs collected on plates were clearly lower than the quantification carried out by the chemical method (Figure 2, Figure 3 and Figure 4 and Table 4). Moreover, in the fine and coarse fractions of the WWTP_RE1 sampling, almost the same amount of spores/bacterial cells sampled was obtained, although not completely in line with the innovative chemical method utilized.

These results were due to the substantial differences on which the biological quantification was based: the classic plate count method of sampled airborne “bio-particles” exhibited microbial charge detection limits, since it only highlighted the cultivable fraction present, and, in addition, it does not distinguish between viable bacterial cells and spores [44].

Even if fungal spores were not found out at the level of the fine fraction, as reported in Figure 7b, they were present in low abundance. This could be explained by the fractionation and size reduction of air-dispersed fungal spores, as reported in the literature [61]. Airborne spores can suffer the effects of humidity, temperature and atmospheric conditions, showing changes and reduction in shape and size, which, even if minimal, can determine granulometric diameters that belong to the fine (PM < 1) fraction [62].

However, the substantial difference in the bacterial and fungal loads collected between the WWTP_RE1 and WWTP_RE2 samples was also found by the CFU counts. This aspect was highlighted by quantification through the chemical method and was always ascribable to the Saharan dust event that occurred during the second sampling of the WWTP_RE campaign, with a consequent strong increase of coarse fraction. This further result was a confirmation of the sensitivity limit and detection demonstrated by the classic biological counting method, which should always be combined and integrated with other quantification approaches, such as molecular and chemical ones.

### 3.4. Identification of Cultivable Bacteria by 16S rDNA Sequencing and Antibiotic Susceptibility Test

The cultivable bacterial species that were isolated by washing the sampled filters were screened through morphological recognition that was carried out by a stereomicroscope and an optical microscope, and then they were identified by the partial sequencing of the 16S rRNA coding gene, which is commonly used for the study of phylogeny in prokaryotes. Sequences of approximately 1000 bases were obtained and compared with GenBank database from National Center of Biotechnology Information through the basic local alignment search tool (BLAST). About fifty colonies with different colors and morphologies, including bacteria (70%), molds, and yeasts (30%), were collected. The isolated cultivable bacteria, identified by 16S rDNA sequencing, are reported in Table 5. As expected, the cultivable bacteria were only a minor fraction of the bacterial populations that were found with the culture-independent analysis. Nevertheless, both approaches indicated that bacteria mainly belonged to the three phyla of Firmicutes, Actinobacteria and Proteobacteria. The most frequently detected genera were *Staphylococcus*, *Micrococcus*, *Bacillus*, *Stenotrophomonas*, *Acinetobacter* and *Moraxella.* Most of them were also detected by the molecular analysis that, interestingly, indicated that *Bacillus* and *Acinetobacter* were among the most abundant populations found.

These data were also supported by others studies in which an identification of airborne microorganisms during an African dust event was carried out [47,63]. In particular, Federici et al. [47] also isolated several species of *Bacillus*, *Pseudomonas* and *Sphingomonas*, while Polymenakou et al. [63] found *Sphingomonas* spp., Micrococcaceae family representatives, and *Acinetobacter* spp. (in particular *Acinetobacter iwoffii*) among the pathogenic species. *Acinetobacter iwoffii* is known as an animal and human opportunist pathogen that has been involved in cases of meningitis and nosocomial infections [64,65]. Another isolate was *Micrococcus luteus* (Table 5), a Gram-positive bacterium that has been implicated in endocarditis and pneumonia cases [66,67]. In order to evaluate the biological risk assessment represented by these two isolates, an antibiotic susceptibility test was performed, and its results are reported in Table 6. Antibiotic susceptibility profiling was determined by the disc diffusion method, and twenty antibiotics, including inhibitors of cytoplasmic membrane function and protein, nucleic acid and cell wall synthesis, were assayed. *A. iwoffii* showed a broader antibiotic resistance profile than *M. luteus*, which only grew in the presence of fosfomycin and aztreonam. The antibiotic resistance analysis was performed on the all isolated species; these data are reported in Table S1.

### 3.5. In Vivo Assay

The nematode *C. elegans* was the in vivo-system model that was chosen for the validation of the pathogenicity of the two isolates *A. iwoffii* and *M. luteus*. These isolates belonging to these species were chosen because their pathogenic behavior in *C. elegans* has not been characterized, unlike to the isolates belonging to species such as *Stenotrophomonas maltophilia* or *Staphylococcus epidermidis*.

**Figure 8:**
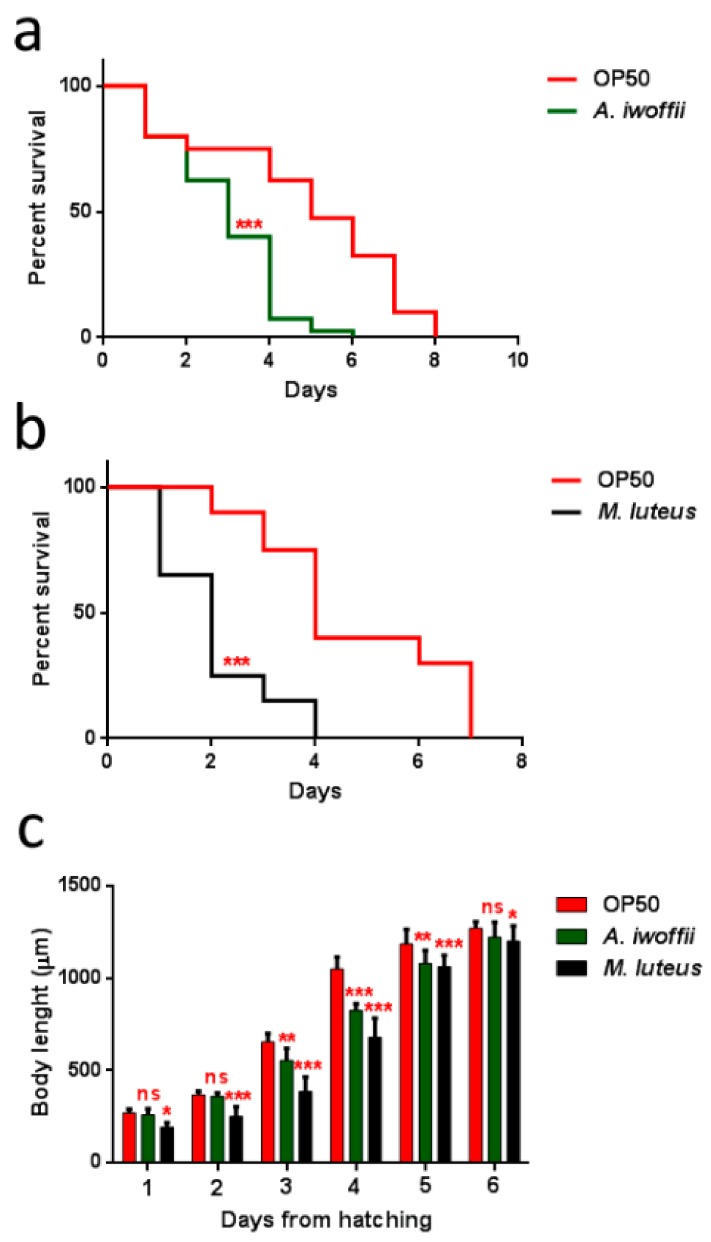
Effects of *M. luteus* and *A. iwoffii* on *Caenorhabditis elegans* physiology. (**a**) Kaplan–Meier survival plot of N2 worms fed with *A. iwoffii* and (**b**) *M. luteus*. Infections were performed at 25 °C, and worm mortality was monitored every day. The lifespan of OP50-fed animals is reported as control; *n* = 60 for each data point of single experiments. Statistical analysis was evaluated by Log-rank (Mantel–Cox) test; asterisks indicate significant differences (*** *p* < 0.001). (**c**) Measurement of worm’s body length starting from their hatching on plates that were seeded with the indicated bacteria. Worm length was measured from head to tail at the indicated time points. Statistical analysis was evaluated by a one-way ANOVA with the Bonferroni post-test; asterisks indicate significant differences (*ns* as non-significant; * *p* < 0.05; ** *p* < 0.01; *** *p* < 0.001).

Infection assays were performed by measuring the survival of animals fed with these bacteria strains from the one-day-adult stage. As shown in Figure 8, both bacteria created a significant *C. elegans* lifespan reduction with respect to the control animals fed with the standard food *E. coli* OP50. The Gram-positive *M. luteus* were already more virulent than *A. iwoffii* starting from the second day of infection, where almost 50% of infected nematodes were dead (Figure 8b). Instead, in the Gram-negative *A. iwoffii* infection, most of the animals’ population died after three days of exposure (Figure 8a). Afterwards, the larval development of nematodes grown on the two isolates was investigated (Figure 8c): Histograms revealed a slower growth for *M. luteus-* and *A. iwoffii-*infected larvae compared to the OP50-fed ones. Additionally in this case, *M. luteus* proved to be more pathogenic than *A. iwoffii*, postponing complete development, which was reached two days after that of the controls.

**Figure 9:**
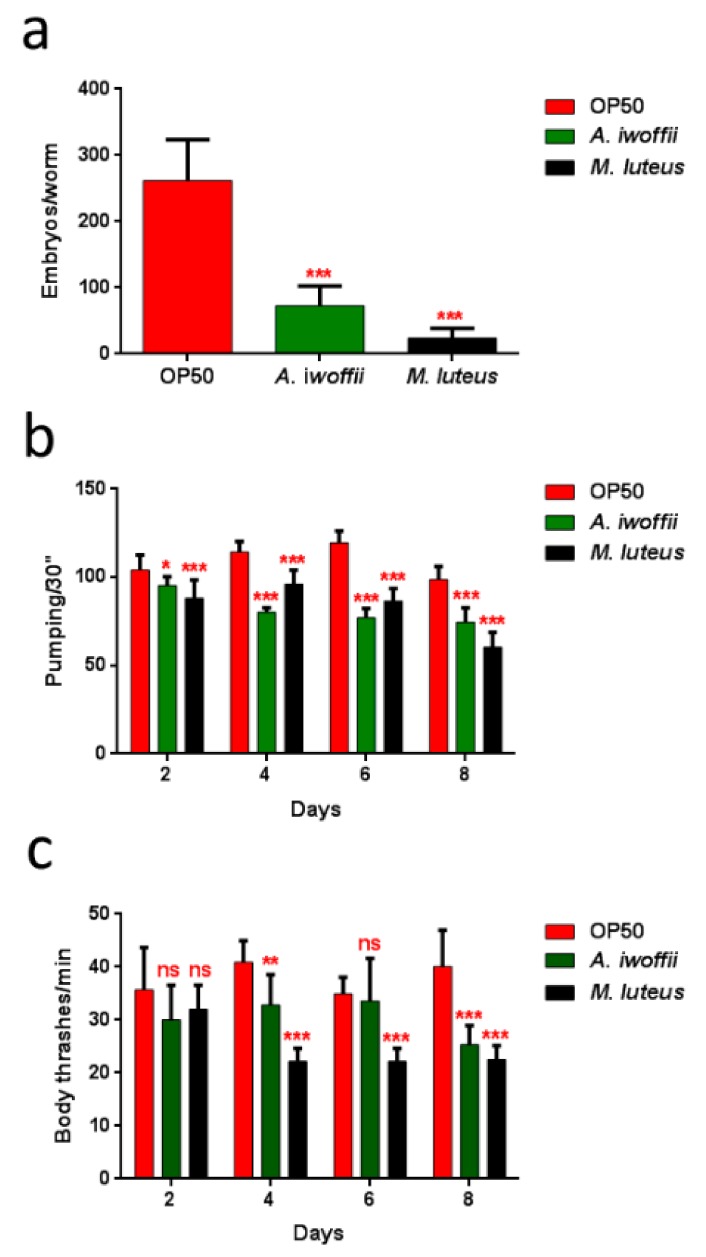
(**a**) Average embryos production per worm of *M. luteus*-, *A. iwoffii*- and OP50-fed animals. Bars represent the standard deviations. (**b**) Pharyngeal pumping rate after continued exposure to the indicated bacteria and worms fed with OP50 were used as controls. The contractions were measured for 30 s and determined from the mean of 10 worms for each bacterial strain. (**c**) Body bend frequency of worms fed with OP50, *M. luteus* and *A. iwoffii* from one-day-adult stage. The number of thrashes was measured in a time period of 60 s. Statistical analysis was performed by a one-way ANOVA with the Bonferroni post-test; asterisks indicate significant differences (ns as non-significant; * *p* < 0.05, ** *p* < 0.01, *** *p* < 0.001).

Moreover, the impact of bacterial infection on *C. elegans* progeny production was evaluated (Figure 9a). Adults fed with isolates exhibited an evident decrease of embryo laying as compared to the *E. coli* OP50-fed controls. The nematodes with the *M. luteus* diet displayed the lowest number of embryos per worm, 10% with respect to the control. Whereas the eggs laid by *A. iwoffii* fed-animals were about 28% compared to the control. Tests regarding involvement in neuronal and motile apparatuses and the pharyngeal pumping rate (Figure 9b) were performed, as were body bending assays (Figure 9c). *M. luteus-* and *A. iwoffii*-fed nematodes showed a reduced pumping rate and a lowering motility than the control worms starting from the second and the fourth day of adulthood, respectively. A strong decrease of these parameters was observed in the isolate-fed nematodes.

A shortened lifespan, a delayed larval development, and a limited pharyngeal pumping rate with a lowered body bending were all evident signs of a bacterial infection, proving that these two isolates have pathogenic behaviors [39,68].

Thus far, no *C. elegans* in vivo-studies concerning these two bacteria have been reported in the literature, but their potential pathogeny for humans is well known. Bacteremia and infections caused by *A. iwoffii* and *M. luteus* are mostly registered as healthcare-associated infections [69,70,71,72]. In comparison with other microorganisms, the two isolates showed certain pathogenic behaviors because they caused all the typical symptoms and phenotypes of a *C. elegans* infection, as reported by Ewbank and Pujol [73]. At the moment, we cannot discriminate if the pathogenic mechanisms of the two bacteria act at the cellular level by colonizing the intestinal lumen (we did not observe the dilatation of nematode intestine) or if they affect *C. elegans’* vitality because of the production of some toxins. These aspects require further investigation in the future. Indeed, due to the ubiquity and dissemination of *A. iwoffii* and *M. luteus* as normal components of human skin flora, more studies based on their danger assessment should be also fundamental.

Though the choice of only two isolates to be analyzed from a pathogenic point of view in vivo seems limited, these results open the exploitation of *C. elegans* as a suitable system for pathogen risk assessment.

## 4. Conclusions

The present work highlights that an integrated multidisciplinary approach is necessary to fully evaluate bioaerosol microbiological richness, which is underestimated by traditional cultural methods. Despite this, the current system allows a rapid and qualitative analysis of the sampling bioaerosol. Chemical and other culture-independent approaches can fill in the gaps of cultural methods. Our chemical approach showed a much higher microbial concentration with respect to the biological method count, since, in addition to alive (viable) microbes, it took not viable, dead organisms, and fragments into account. Besides, the sequencing of 16s rRNA encoding gene starting from the extracted DNA of picked bacterial colonies showed a scarce and limited identification of the species when compared with that obtained by NGS analysis. Two of bacterial isolates found in this bioaerosol sampling that can cause human diseases and infections were resistant to more than one antibiotic molecule, and the possible danger they represented was assessed on a *C. elegans* model system. The identification and quantification of these species should be supported by biological, chemical, and molecular methods, combining and confronting all together the aspects. This could lead a complete and precise assessment of bioaerosol exposures in occupational environments in order to protect workers’ health.

## Figures and Tables

**Table 1:** Sampling campaigns performed in the wastewater treatment plants (WWTPs), located in central Italy.

Date	Acronym	Da (µm)	Volume m^3^	PM (µg/m^3^)
5–11/04/18	WWTP_RE1	<1	84	10
		1–10	84	13
11–16/04/18	WWTP_RE2	<1	72	12
		1–10	72	37

Da: aerodynamic diameter. Volume m^3^: volume of sampled air. PM: particulate matter.

**Table 2:** Standard and reagent brands.

Standards and Reagents	Company
Acetonitrile (AcN)	Romil LTD (Cambridge, UK)
Methanol (MeOH	Romil LTD (Cambridge, UK)
Ethanol (EtOH)	Romil LTD (Cambridge, UK)
Dichloromethane (DCM)	Romil LTD (Cambridge, UK)
2,2,4-Trimethylpentane (iso-octane, ISO)	Romil LTD (Cambridge, UK)
Propan-2-ol (IPA)	Romil LTD (Cambridge, UK)
Water (H_2_O)	Romil LTD (Cambridge, UK)
Hydrochloric acid (HCl) 30%	Merck S.p.a. (Vimodrone, Milan, Italy),
2,6-Pyridinedicarboxylic acid (Dipicolinic acid, DPA)	Sigma-Aldrich S.r.l. (Milan, Italy)
Muramic acid (MUR)	Sigma-Aldrich S.r.l. (Milan, Italy)
Ergosterol (ERG)	Sigma-Aldrich S.r.l. (Milan, Italy)

**Table 3:** Instrumentation.

Analyte	Column	Brand	HPLC	MS-MS	Software
MUR DPA	SeQuant^®^ ZIC^®^- HILIC column (5 µm polymer 150 × 2.1 mm) + guard column (20 × 2.1 mm)	Merk s.p.a (Vimodrone, Milan, Italy).	1260 Infinity II system (Agilent Technologies Italy S.p.A. Cernusco sul Naviglio MI, Italy)	API 2000 + ESI source (AB SCIEX S.r.l. Forster City, CA, USA)	Analyst 1.6.2
ERG	Discovery C_8_ (50 × 2.1 mm, 5 µm) + guard column (20 × 2.1 mm)	Phenomenex (Torrance, CA, USA)	1290 Infinity system (Agilent Technologies Italy S.p.A. Cernusco sul Naviglio MI, Italy)	G 6460 +APCI source (Agilent Technologies Italy S.p.A. Cernusco sul Naviglio MI, Italy)	Mass Hunter Workstation software B.06.00

**Table 4:** Summary of microorganisms number/m^3^ obtained during the monitoring campaign in particulate matter (PM) < 1 and PM > 1 dimensional fractions.

	n° Microorganisms/m^3^	WWTP_RE1	WWTP_RE2
**Bacterial Cells**	PM < 1	2.10 × 10^5^	1.70 × 10^5^
	PM > 1	7.20 × 10^4^	4.70 × 10^5^
**Bacterial Spores**	PM < 1	1.10 × 10^5^	1.00 × 10^5^
	PM > 1	1.70 × 10^4^	4.60 × 10^4^
**Fungal Spores**	PM < 1	* n.d.	* n.d.
	PM > 1	2.90 × 10^3^	1.10 × 10^4^

* n.d. not detected.

**Table 5:** List of isolated-cultivable bacteria derived from washing the sampled filters. The basic local alignment search tool (BLAST) percent identity expresses how similar the query sequence (rDNA16S) of each isolate was to the target sequence that was present in the database.

Isolate Number	Strain ID	Bacterial Species	BLAST Percent Identity	GenBank Accession Number
1	AW 25	*Staphylococcus warneri*	99%	NR_025922.1
2	CMS 76or	*Kocuria polaris*	98%	NR_028924.1
7	LMG 7040	*Pseudomonas oryzihabitans*	99%	NR_117269.1
8	C58	*Agrobacterium fabrum*	98%	NR_074266.1
9	ZS207	*Acinetobacter iwoffii*	93%	CP019143.1
10	TA68	*Kocuria rhizophila*	99%	NR_026452.1
12	NBRC 12092	*Bacillus pumilus*	99%	NR_112637.1
13	DSM 11821	*Bacillus mycoides*	99%	NR_024697
14	DSM 13	*Bacillus licheniformis*	99%	NR_118996.1
15	DSM 6998	*Moraxella osloensis*	98%	NR_113392.1
16	DM 122	*Staphylococcus hominis*	99%	NR_036956.1
18	ODN7	*Sphingomonas hankookensis*	98%	NR_116570.1
19	G2-1	*Paenarthrobacter nitroguajacolicus*	99%	NR_027199.1
20	NCTC 2665	*Micrococcus luteus*	99%	NR_075062.2
23	ICB 89	*Stenotrophomonas pavanii*	99%	NR_116793.1
29	DSM 20578	*Microbacterium oxydans*	99%	NR_044931.1
30	IAM 12423	*Stenotrophomonas maltophilia*	99%	NR_041577.1
32	A1920	*Moraxella osloensis*	98%	NR_104936.1
33	P 369/06	*Microbacterium phyllosphaerae*	99%	NR_025405.1
35	LMG 25348	*Stenotrophomonas pavanii*	99%	NR_118008.1
36	B6	*Aquabacterium parvum*	95%	NR_024874.1
38	Fussel	*Staphylococcus epidermidis*	99%	NR_036904.1
40	AE-6	*Micrococcus aloeverae*	99%	NR_134088.1

**Table 6:** Antibiotic resistance of isolated *Micrococcus luteus* and *Acinetobacter iwoffii*. The table shows resistances (R) and inhibition zones with relative measures in centimeters (from the center of the disc) for each tested molecule. R means that no inhibition zone was detected.

Antibiotics	μg	*A. iwoffii*	*M. luteus*
Ampicillin	10	R	5 cm
Tetracycline	30	2.5 cm	4 cm
Chloramphenicol	30	4 cm	4.5 cm
Erythromycin	15	3.5 cm	3 cm
Cephalothin	30	R	3 cm
Clindamycin	2	R	3 cm
Cefotaxime	30	R	3 cm
Cefuroxima	30	R	3 cm
Rifamycin	30	2 cm	3 cm
Oxacillin	1	R	1.5 cm
Mezlocillin	75	1 cm	3 cm
Amikacin	30	1 cm	1.5 cm
Fosfomycin	50	R	R
Aztreonam	30	1 cm	R
Gentamycin	10	1 cm	2 cm
Tobramycin	10	1 cm	1.5 cm
Carbenicillin	100	1 cm	3 cm
Streptomycin	25	2 cm	3 cm
Penicillin	10 u	1 cm	4 cm
Vancomycin	30	1 cm	3 cm

## Supplementary Materials

The following are available online at https://www.mdpi.com/1660-4601/17/1/273/s1, Table S1: Antibiotic resistance of the all isolates.
