# Supplementary material for: Evaluation of Bioaerosol Bacterial Components of a Wastewater Treatment Plant Through an Integrate Approach and In Vivo Assessment
Source: Int J Environ Res Public Health. 2019 Dec 30;17(1):273. doi: 10.3390/ijerph17010273 (PMC6981557; doi:10.3390/ijerph17010273)
Supplement: Supplementary file 1 [file ijerph-17-00273-s001.pdf]

Table S1. Antibiotic resistance of the all isolates. Table shows inhibition zones with relative measures in centimeters (from the center of the disc) for each tested molecule. R means that no inhibition zone was detected.

**Antibiotics**																				
**Isolates**	**AM**	**TE**	**C**	**E**	**KF**	**DA**	**CTX**	**CXM**	**RA**	**OX**	**MEZ**	**AK**	**FF**	**ATM**	**CN**	**TOB**	**PY**	**S**	**P**	**VA**
Staphylococcus warneri	1.5	2.5	2.5	2	2.5	R	1	3	4	2	1	1	R	R	1	1	1	1	R	2
Kocuria polaris	1.5	2	2.5	2	1.2	R	R	R	2	R	2	3	R	R	2	1	R	3	2	2
Pseudomonas oryzihabitans	1.5	2	2.5	R	R	R	1	R	R	R	2	1	R	1	1	1	1	2	1	R
Agrobacterium fabrum	R	2.5	1	R	R	R	R	R	2	R	R	1	R	R	1	1	R	2	R	1
Acinetobacter iwoffii	R	2.5	4	3.5	R	R	R	R	2	R	1	1	R	1	1	1	1	2	1	1
Kocuria rhizophila	R	1	2	R	1	R	2	1	1	R	R	2	R	1	1	1	1	3	2	2.5
Bacillus pumilus	2.9	2.9	2.5	2.5	2.8	1	R	R	2	1	2.5	2	R	R	2	1	2	2	2	2
Bacillus mycoides	1.5	2	3	2.5	3.5	1	R	R	1.5	3	2	1.5	R	R	1.5	3	1	1	1	1
Bacillus licheniformis	1.6	3.2	1.7	3.6	1.2	R	R	1	4	2	2	1	R	R	2	2	1	2	1	2
Moraxella osloensis	3	3	4.5	2	2	R	2.5	3	3	R	2.5	1.5	R	2.5	1	1	2	2	2	R
Staphylococcus hominis	1.5	3	4	3	R	3	2	3	4	4	2	1	R	R	2	1	1	2	1	2
Sphingomonas hankookensis	R	4	4	3.5	R	3	2	2	2	R	2	2	1	3	1	2	2	2	2	2
Paenarthrobacter nitroguajacolicus	3	2.5	3	3	2	2	2	2	1	2	3	1	R	R	2	1	2	2	4	2
Micrococcus luteus	5	4	4.5	3	3	3	3	3	3	1.5	3	1.5	R	R	2	1.5	3	3	4	3
Stenotrophomonas pavanii	R	1.3	2	R	R	1	1	1.5	1	R	1.1	1	2	R	2	1	R	1	2	2.2
Microbacterium oxydans	1.5	2.7	2.5	1.8	2	3	R	1	3	3	R	1	R	R	1	1	R	2	R	1
Stenotrophomonas maltophilia	R	1.3	2	R	R	R	R	R	R	R	R	R	R	R	R	1	R	R	R	1.5
Moraxella osloensis	2	2.5	3.5	1.8	1.5	3	2	2	2	2	2	2	R	R	2	1	2	2	1	2
Microbacterium phyllosphaerae	1	R	1.5	1	R	R	R	R	1	R	2	1	R	R	R	R	R	1	2	1
Stenotrophomonas pavanii	R	1	2	R	R	R	R	R	R	R	R	1	R	R	R	R	R	1	R	R
Aquabacterium parvum	1.3	1.9	1	1	R	R	R	R	1	R	2	1	R	R	R	R	R	1	2	2
Staphylococcus epidermidis	3	3	4	2	1	3	3	3	3	4	3	3	R	R	2	3	2	3	3	3
Micrococcus aloeverae	4	4	5	3.5	2.6	3	2	2	3	1	2	2	R	R	1	1	2	2	1	2

AM, Ampicillin; TE, Tetracycline; C, Chloramphenicol; E, Erythromycin; KF, Cephalothin; DA, Clindamycin; CTX, Cefotaxime; CXM, Cefuroxima; RA, Rifamycin; OX, Oxacillin; MEZ, Mezlocillin; AK, Amikacin; FF, Fosfomycin; ATM, Aztreonam; CN, Gentamycin; TOB, Tobramycin; PY, Carbenicillin; S, Streptomycin; P, Penicillin; VA, Vancomycin.
